# REM sleep behavior disorder (RBD): Update on diagnosis and treatment

**DOI:** 10.1007/s11818-016-0048-6

**Published:** 2016-04-28

**Authors:** Birgit Högl, Ambra Stefani

**Affiliations:** Department of Neurology, Medical University of Innsbruck , Anichstraße 35, 6020 Innsbruck, Austria

**Keywords:** Parasomnias, Violent dream, REM sleep, Polysomnography, SINBAR, Parasomnien, Gewalt im Traum, REM-Schlaf, Polysomnographie, SINBAR

## Abstract

**Background:**

REM sleep behavior disorder (RBD) is parasomnia characterized by dream enactment and enabled by disruption of physiological muscle atonia during REM sleep. Over the past few years, diagnostic criteria and the methods used to confirm diagnosis have been updated.

**Objective:**

In this review article, the current knowledge regarding RBD diagnosis and treatment is presented.

**Methods:**

A selective literature search was carried out.

**Results and discussion:**

Although several RBD screening questionnaires have been developed, diagnosis can only be definitely confirmed on the basis of polysomnography. New methods for scoring electromyography (EMG) activity during REM sleep have been proposed during recent years and cutoff values have been established. The latest cutoff values for scoring EMG activity during REM sleep are included in the International Classification of Sleep Disorders (ICSD). The cutoff of 27 % muscle activity during REM sleep suggested by the Sleep Innsbruck Barcelona (SINBAR) group was also included in the third edition of the ICSD. The best-researched treatments for RBD are clonazepam and melatonin.

## Zusammenfassung

**Hintergrund:**

Die REM-Schlaf-Verhaltensstörung (REM Sleep Behavior Disorder, RBD) ist eine Parasomnie, die durch Ausagieren von Träumen gekennzeichnet ist und durch eine Störung der physiologischen Muskelatonie des REM-Schlafs ermöglicht wird. In den letzten Jahren wurden die diagnostischen Kriterien und die Methoden zur Diagnosestellung aktualisiert.

**Ziel der Arbeit:**

In diesem Übersichtsartikel werden die derzeitigen Erkenntnisse zur Diagnose und Therapie vorgestellt.

**Methode:**

Es wurde eine selektive Literaturrecherche durchgeführt.

**Ergebnisse und Diskussion:**

Obwohl mehrere Fragebögen zum Screening für RBD entwickelt wurden, kann eine sichere Diagnose nur anhand einer Polysomnographie gestellt werden. Neue Methoden zur Auswertung der EMG-Aktivität im REM-Schlaf wurden in den letzten Jahren vorgeschlagen und Cut-off-Werte wurden etabliert. In die International Classification of Sleep Disorders sind die neuesten Cut-off-Werte für Scoring von EMG-Aktivität im REM-Schlaf eingegangen. Der von der SINBAR (Sleep INnsbruck BARcelona)-Gruppe vorgeschlagene Cut-off von 27 % Muskelaktivität im REM-Schlaf ist auch in die ICSD-3 übernommen worden. Die am besten untersuchten Therapien bei RBD sind Clonazepam und Melatonin.

## Background

The International Classification of Sleep Disorders (ICSD-3) [1] states the following diagnostic criteria for REM sleep behavior disorder (RBD): (1) Repeated episodes of sleep-related vocalization and/or complex motor behaviors. (2) These behaviors are documented by polysomnography to occur during REM sleep or, based on clinical history of dream enactment, are presumed to occur during REM sleep. (3) Polysomnographic recording demonstrates REM sleep without atonia (RWA). (4) The disturbance is not better explained by another sleep disorder, mental disorder, medication or substance abuse.

These criteria ensure that definitive diagnosis of RBD according to the ICSD can only be made on the basis of polysomnography (PSG). Exactly how RBD is diagnosed using PSG will be discussed later.

This review article presents the current knowledge pertaining to diagnosis and treatment of RBD.

## Clinical picture

The prevalence of RBD is reported to be 0.38–2.1 % [2, 3] in the general population. Prevalence rates are higher among patients with Parkinson’s disease (PD) or other synucleinopathies: 51 % among patients with de novo PD [4] and up to 88 % among patients with multiple system atrophy (MSA) [5–8] and various other diseases [9].

Particularly characteristic of RBD is that patients enact their dreams using movements and vocalizations. This often gives patients’ bed partners the impression that they know what is happening in the dream, for example if the patient is trying to chase away a dog by kicking their feet and cursing loudly. Also characteristic is that the RBD episodes, which are associated with REM sleep, normally arise after midnight and generally do not occur during the first hour after falling asleep. If patients are woken during an RBD episode, they can often report on an elaborate dream. Patients are generally easy to wake and quick to reorient themselves. One further characteristic is that the behavior exhibited during an RBD episode is highly variable, even when the same patients are considered.

## Screening methods

Since polysomnographic evaluation is not universally available and diagnosing RBD requires specific qualifications, numerous questionnaires have been developed to screen for RBD. It is important to note that questionnaires only enable diagnosis of probable RBD.

The first and most frequently applied questionnaire was devised by Karin Stiasny-Kolster and published in 2007 [10]. This questionnaire comprises 10 items, which are answered by 13 “yes” or “no” forced-choice questions. The Hong Kong Questionnaire [11] was developed three years later and comprises 13 questions assessing symptoms which have arisen during the patient’s lifetime, as well as the frequency of these symptoms during the past year. The latter questionnaire also features a “don’t know” option. The Mayo Sleep Questionnaire [12, 13] is not exclusively dedicated to RBD, but does include an introductory RBD question, which, if positive, leads on to further questions. The Innsbruck RBD Inventory [14] is a simple questionnaire containing only five specific RBD questions, which can be answered with “don’t know” as well as with “yes” or “no”.

In addition, two single questions have been developed for RBD screening purposes. The first of these is RBD1Q, published by Ron Postuma and International RBD Study Group authors [15]. The single question is as follows: “Have you ever been told, or suspected yourself, that you seem to ‘act out your dreams’ while asleep (for example, punching, flailing your arms in the air, making running movements, etc.)?” The Innsbruck RBD Inventory also includes a single question for screening for RBD: “Do you kick or hit during your sleep because you dream that you have to defend yourself?” [14]. All of the aforementioned questionnaires have been validated and demonstrated acceptable sensitivity and specificity in the validation studies [16].

Nevertheless, recent experiences have shown that uncritical use of questionnaires can lead to false-negative and false-positive results, particularly if patients complete them alone and without the help of a trained interview partner: a striking observation was that healthy individuals, with no indications of RBD in a subsequent sleep interview and PSG examination, scored 16 % false-positive on the RBD Inventory [17]. It has also been revealed that the prevalence of probable RBD in population studies varies when several questionnaires are used in parallel [18]. Also, among PD patients, the number of diagnoses correctly identified using questionnaires differs widely from subsequent PSG, depending on the settings under which the questionnaire was applied [19].

## What role does video play in the diagnosis of RBD?

During the past decade, numerous authors have concerned themselves with the analysis of video recordings of RBD patients. Video analysis methods ranged from description only to a severity classification [20]. To the best of our knowledge, the first study on this topic was performed by Emilia Sforza in 1988 [21]. Over the past 10 years, our group has also worked on video classification of motor events in RBD patients [22–24]. We were able to show that even among patients with severe RBD, the majority of motor events are “very small elementary movements.” The far better-known dramatic, “violent” behaviors are comparatively rare, even in severe RBD, and should as such be interpreted as the proverbial “tip of the iceberg.” Furthermore, we were able to demonstrate that the majority of elaborate and violent motor events were initiated during REM sleep with rapid eye movements (in contrast to REM sleep without rapid eye movement), such that one can speak of a gating function of REM sleep with rapid eye movements. Simple myoclonic background jerking is, however, observed during the entire phase of REM sleep.

In contrast to the very elaborate approach of descriptive, videography-based characterization of RBD events, Sixel-Döring and Trenkwalder have developed a very simple severity scale for clinical routine [25]. Using this scale, motor events are classified from 0 to 3 (0: REM only without atonia, 1: small distal movements, 2: proximal muscle involvement, 3: with axial movements; vocalization is classified as “1” present or “0” absent). The same working group also showed that in newly diagnosed, as-yet-untreated PD patients without RBD, very small motor events (REM sleep behavioral events, RBE) possibly precede the diagnosis of full-blown RBD, and could thus perhaps serve as early markers of neurodegeneration [26].

## Video-polysomnography and EMG analysis

While video analyses depend on the occurrence of unpredictable, perhaps rare events, polysomnographic EMG analysis has the advantage that REM sleep without atonia exhibits very high night-to-night stability. Several studies have demonstrated that even a single polysomnography night is adequate (providing REM sleep is present) for diagnosis of RBD [27, 28]. Moreover, polysomnography also has the advantage that the investigator can select which muscle channels are registered in addition to the EEG, EOG, and cardiorespiratory channels. The selection ranges from the classic PSG muscles (mental, submental, and tibialis anterior muscles) to numerous other muscles of the upper and lower extremities, proximal and distal, agonists and antagonists, as well as muscles of the trunk or other muscles served by cranial nerves [29, 30].

**Fig. 1:**
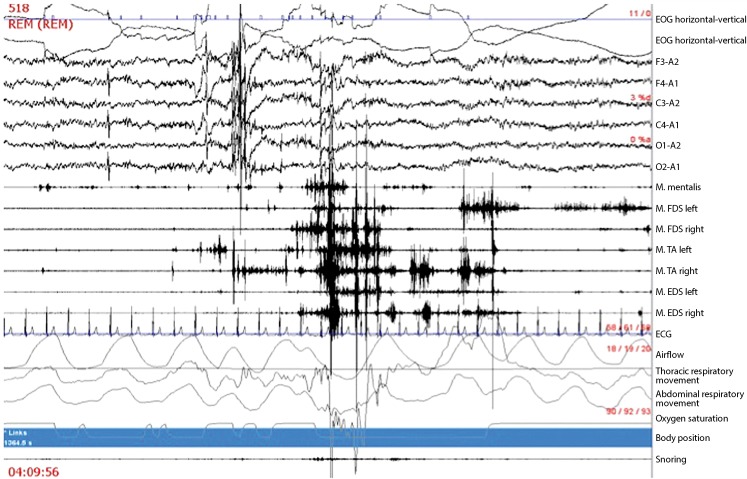
30-s epoch of REM sleep, with REM sleep without atonia in the seven muscle channels between EEG and ECG

**Fig. 2:**
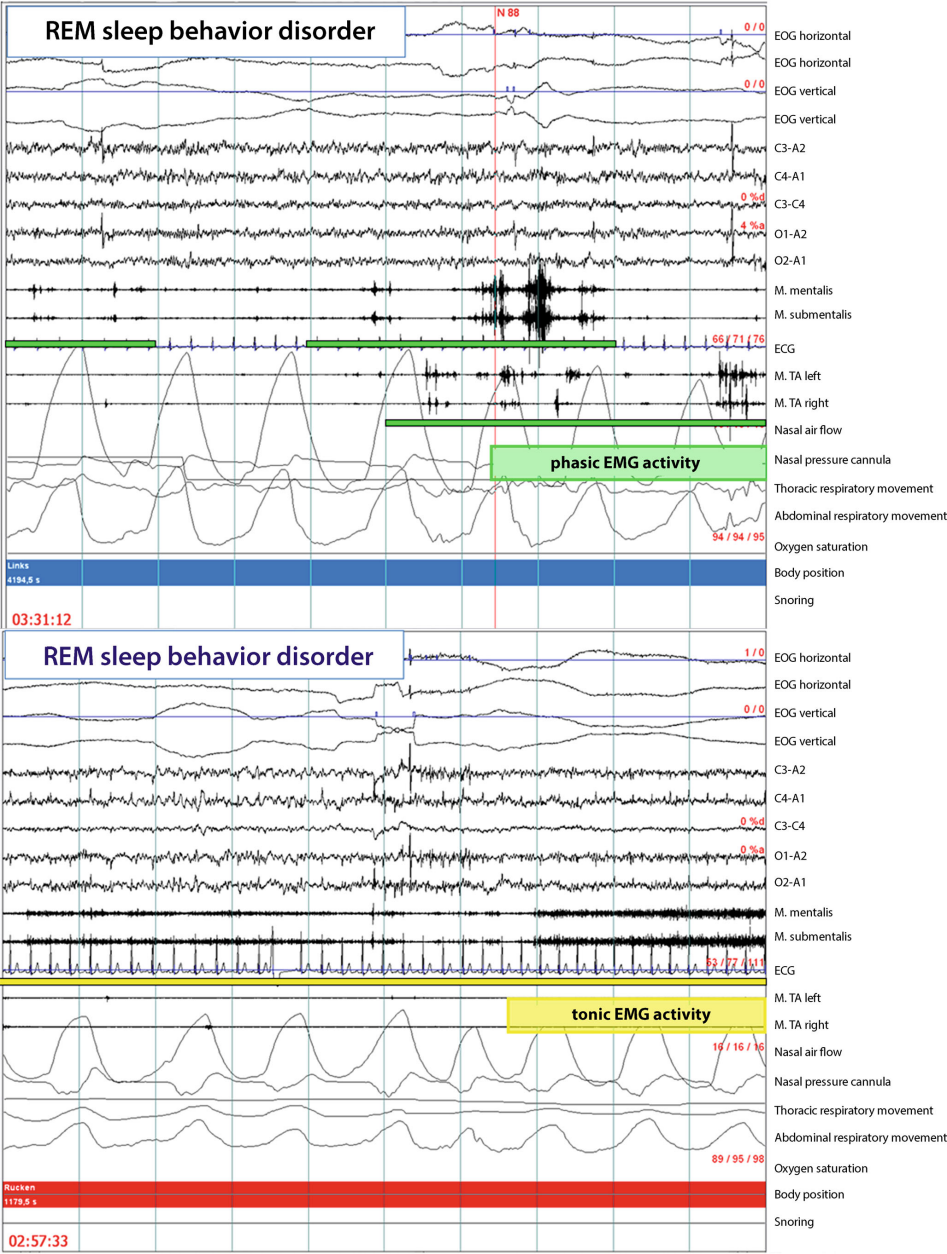
Phasic and tonic EMG activity in one epoch of REM sleep in RBD patients

The characteristic finding in RBD patients is increased muscle activity during REM sleep, which is frequently immediately recognizable, particularly when enough EMG channels are registered. The current scoring methods are based on differentiation between tonic and phasic muscle activity, as originally described by Lapierre and Montplaisir [31]. Various other designations and additional criteria have, however, been proposed (see [20] for a review). The Sleep Innsbruck Barcelona (SINBAR) group performed multiple investigations aimed at determining which minimal combination of EMG channels/muscle registrations permitted reliable diagnosis of RBD. Following several initial studies [29, 32], it was also possible to publish normative values for the first time, above which detected tonic and phasic muscle activity (defined in Tab. 1 and presented exemplarily in Fig. 1 and 2) can be viewed as RBD, provided the other diagnostic criteria, i. e., clinical or videographic criteria, are fulfilled. The latter study also demonstrated that chin muscle registrations for RBD diagnosis are especially well complemented by registration of the flexor digitorum superficialis muscle in the forearm, since muscle activity during REM sleep in this region is particularly specific to RBD. In contrast, the frequently registered tibialis anterior muscle is significantly less specific, particularly in light of the fact that patients in the second half of their lives frequently also exhibit pathological muscle activity during REM sleep in this area due to other factors, e. g., neuropathies or radicular lesions. Additionally, it was observed that tonic EMG activity, which is only measured on the chin, and phasic muscle activity can be meaningfully extended by an “any” EMG activity category: based on the previous differentiation, all muscle activities lasting between 5 and 15 s (with 30-s epochs), i. e., everything which did not correspond to the criteria for tonic (>15 s) or phasic (0.1-5 s) activity, was not counted. It is important to note that EMG analysis usually takes place in 3‑s mini-epochs. This means that, for example, a phasic switch in one out of ten mini-epochs would correspond to 10 % RWA-positive mini-epochs. Using this method it could be shown that, compared to controls, far more EMG activity was found in RBD in every single one of the 13 striated muscles investigated (cranial nerve supplied, upper and lower extremities [30]), although combination of the mentalis and the flexor digitorum superficialis muscles proffered the best sensitivity and specificity with minimal registration channels. Where RBD is suspected it is therefore recommended – and this is in agreement with recommendations made by Mahowald and Schenck 30 years ago [33] – that the upper extremities also be registered during polysomnography. By applying the SINBAR methods [30], cutoff values could also be published for the first time (for the chin 3‑s mini-epochs: “any” EMG activity at 18 %; for the combination of chin and flexor digitorum superficialis: the cutoff value was 32 % based on entire REM sleep for the 3‑s mini-epochs, and 27 % for 30-s epochs according to a simplified analysis based on American Academy of Sleep Medicine recommendations). Similar normative values have since been published by the Rochester group [34].

**Tab. 1:** Definition of phasic, tonic, and “any” EMG activity during REM sleep according to the SINBAR criteria [30]

**EMG activity in REM sleep**	
Phasic	Short burst of EMG activity lasting between 0.1 and 5 s, which is more than twice as high as the background EMG amplitude. Can be measured in 3‑s mini-epochs or 30‑s epochs
Tonic	EMG tone increased by at least a factor of two or four compared to baseline in more than half of the epoch, i.e. ≥ 10 s in 20‑s epoch or ≥ 15 s in 30‑s epoch.
“Any”	Either phasic or tonic EMG activity. In addition, tonic and phasic muscle activity lasting between 5 and 15 s can be scored. Can be measured in 3‑s mini-epochs or 30‑s epochs

*EMG* electromyography, *REM* rapid eye movement

The disadvantage of manually quantifying EMG activity during polysomnography is that it is hugely time intensive and places high technical demands on the scorer for this type of analysis. For this reason, many investigations aimed at achieving computer-assisted quantification of EMG activity during REM sleep have been carried out during the past 10 years. The earliest attempts were made in the USA; later on, programs from Germany, Denmark, Italy, and Austria were also validated [16]. These programs were based on very different algorithms. The algorithm used in our clinic is based on an algorithm built into the PSG system, which enables tonic and phasic muscle activity in the chin and the flexor digitorum superficialis (as well as in other, freely programmable muscles) to be precisely quantified according to the well-defined criteria “tonic”, “phasic”, and “any”. This method has been validated and demonstrated excellent results [35]. One of the better-known analysis methods is the REM atonia index, which was developed by Raffaele Ferri and considers only the activity of the chin musculature. The latter analysis requires an additional program [36, 37].

Despite the promise of simple indices and computer-assisted quantification methods for REM sleep without atonia, solid analysis of the raw data with precise elimination of artefacts is essential to avoid false positives (e. g., false-positive RWA classification in patients with snoring or movement artefacts accompanying respiratory events) [35].

## RBD as an early indicator of neurodegenerative disease

Studies on RBD as an early indicator of neurodegenerative disease published in the 1990s and early 2000s reached the conclusion that approximately 40–50 % of RBD patients would develop a neurodegenerative disease, primarily a synucleinopathy, a good 10 years later [38, 39]. In 2009, the 12-year risk was reported to be 52.4 % [40]. However, current studies have demonstrated that the rate of conversion to a synucleinopathy is much higher, at 81 % [41, 42]. In light of this high conversion rate and the long time interval between diagnosis of RBD and conversion, the question obviously arises of whether other biomarkers exist, ones which are able to indicate an increased risk of conversion in the near future. This predictive property has been investigated for numerous risk markers (particularly anosmia/hyposmia, autonomic dysfunction, neuropsychologic tests, color discrimination, and quantitative motor tests). These studies showed, for example, that in patients with RBD still classified as idiopathic, the coexistence of olfactory disturbance or color vision impairment was associated with an increased risk of conversion to a neurodegenerative disease [43, 44]

Although one thinks primarily of polysomnography for RBD, 1.5 Tesla DTI and VBM MRI also enable excellent discrimination between RBD subjects and controls: in 26 RBD patients and 14 controls, Scherfler and colleagues were able to show that reductions in fractional anisotropy were found in areas such as the periaqueductal grey matter and the lateral pontine tegmentum in RBD patients. Additionally, increased mean diffusivity was observed in RBD patients in areas which could correspond to the sublaterodorsal nucleus, the pedunculopontine nucleus, and the locus coeruleus, as well as in areas important for regulation of muscle tone during REM sleep [45]. Further imaging studies were performed by numerous groups using different methods, some of which revealed involvement of yet other areas [46–48]. Using FP-CIT SPECT and transcranial sonography, it was possible to show – with 100 % sensitivity – that among 43 RBD patients, those individuals who already had an abnormal baseline finding in one of the methods converted to a neurodegenerative disease within 2.5 years [49]. Furthermore, a dopamine transporter follow-up study using putamen and caudate measurements also showed that patients who converted had lower baselines and an increased decline in uptake [50].

## New results from long-term studies on surrogate markers of neurodegenerative disease

Mahlknecht and colleagues evaluated IRBD patients using complete Sniffin’ Sticks tests, and were able to show that olfactory function could predict conversion into Lewy body disease [43]. Recently, the same group showed that a subscore of the top 8 discriminating odors was as good as the whole Sniffin’ Sticks test [51]. On the other hand, the SINBAR group was able to demonstrate that sonographically determined changes in the substantia nigra are unsuitable for monitoring the process of neurodegenerative disease, since these are stable over the years [52]. A joint investigation by the International RBD Study Group revealed an unexpected association with lifetime use of antidepressants in patients with IRBD, an effect that was stronger than the association with depression alone. Furthermore, an association with ischemic heart disease was also observed [53].

In a four-year follow-up investigation, the International RBD Study Group evaluated 279 RBD patients from 12 centers who had initially participated in a questionnaire study. Patients who converted were older. Neither caffeine, nor smoking, nor alcohol exposure was able to predict conversion. Unexpectedly, converted patients were less likely to have been exposed to pesticides and were more likely to have a family history of dementia. Motor and autonomic symptoms were also more frequently observed in converted patients, and among converted patients with dementia, clonazepam use was more frequent [54].

## Therapy

The best-investigated substances used to treat RBD are clonazepam and melatonin. Numerous larger series and cohort studies have demonstrated good efficacy of clonazepam 0.5–2 mg [55]. Melatonin 3–9 mg has also been tested many times openly, as well as in one much smaller study with a double-blind design [56]. Large multicenter, double-blind, placebo-controlled studies with well-defined outcomes are urgently needed.

Earlier case studies addressing treatment of REM sleep disorder also mention many other substances, although the significance and validity of these case studies is often questionable – not only due to their open nature and the very small number of cases considered, but also in light of the sometimes disputable RBD diagnosis (particularly where PSG is lacking and the clinical symptoms described are atypical).

## Do RBD patients really dream differently?

Even early studies indicated that PD patients on levodopa treatment exhibited altered dream content, although RBD had not yet been defined at this time [57]. Classically, RBD dreams are described as being particularly “action-packed”, and often involve fight or flight, attacks, or animals [58]. By reading the dream report aloud and subsequently playing different video clips, an earlier study was able to show, with statistical significance, that independent observers can match the behaviors observed in RBD patients to particular portions of the dream better than chance [59]. Recently, Katja Valli was also able to demonstrate that the dreams of PD patients with RBD did not differ greatly from PD patients without RBD when woken directly from REM sleep [60].

## Practical conclusion

- Suspicion of REM sleep behavior disorder can be diagnosed based on medical history and clinical presentation. Questionnaires can be used as a screening method. Definitive diagnosis can only be established using polysomnography.
- Video-polysomnography demonstrates that “violent” behavior is rare, even in severe RBD. The majority of motor events are very small elementary movements.
- The advantage of EMG analysis during polysomnography is that increased muscle activity during REM sleep (REM sleep without atonia), which is characteristic of RBD, has a very high night-to-night stability.
- Specific suitable muscle registrations and cutoff values are available for RBD diagnosis. Recording of forearm FDS muscle is much recommended in addition to the tibialis anterior and mentalis muscles. If EMG activity is approximately 1/3 of REM sleep this is in line with RBD.
- Clonazepam and melatonin are the best-investigated therapeutic agents.

## References

[1] American Academy of Sleep Medicine (2014). International classification of sleep disorders.

[2] Chiu HFK, Wing YK, Lam LCW (2000). Sleep-related injury in the elderly – an epidemiological study in Hong Kong. Sleep.

[3] Kang SH, Yoon IY, Lee SD, Han JW, Kim TH, Kim KW (2013). REM sleep behavior disorder in the Korean elderly population: prevalence and clinical characteristics. Sleep.

[4] Mollenhauer B, Trautmann E, Sixel-Döring F (2013). Nonmotor and diagnostic findings in subjects with de novo Parkinson disease of the DeNoPa cohort. Neurology.

[5] Wetter TC, Collado-Seidel V, Pollmächer T, Yassouridis A, Trenkwalder C (2000). Sleep and periodic leg movement patterns in drug-free patients with Parkinson’s disease and multiple system atrophy. Sleep.

[6] Vetrugno R, Provini F, Cortelli P (2004). Sleep disorders in multiple system atrophy: a correlative video-polysomnographic study. Sleep Med.

[7] Plazzi G, Corsini R, Provini F (1997). REM sleep behavior disorders in multiple system atrophy. Neurology.

[8] Palma JA, Fernandez-Cordon C, Coon EA (2015). Prevalence of REM sleep behavior disorder in multiple system atrophy: a multicenter study and meta-analysis. Clin Auton Res.

[9] Husain AM, Miller PP, Carwile ST (2001). Rem sleep behavior disorder: potential relationship to post-traumatic stress disorder. J Clin Neurophysiol.

[10] Stiasny-Kolster K, Mayer G, Schäfer S, Möller JC, Heinzel-Gutenbrunner M, Oertel WH (2007). The REM sleep behavior disorder screening questionnaire – a new diagnostic instrument. Mov Disord.

[11] Li SX, Wing YK, Lam SP (2010). Validation of a new REM sleep behavior disorder question. Sleep Med.

[12] Boeve BF, Molano JR, Ferman TJ (2011). Validation of the Mayo Sleep Questionnaire to screen for REM sleep behavior disorder in an aging and dementia cohort. Sleep Med.

[13] Boeve BF, Molano JR, Ferman TJ (2013). Validation of the Mayo Sleep Questionnaire to screen for REM sleep behavior disorder in a community-based sample. J Clin Sleep Med.

[14] Frauscher B, Ehrmann L, Zamarian L (2012). Validation of the Innsbruck REM sleep behavior disorder inventory. Mov Disord.

[15] Postuma RB, Arnulf I, Hogl B (2012). A single-question screen for rapid eye movement sleep behavior disorder: a multicenter validation study. Mov Disord.

[16] Frauscher B, Högl B, Videnovic A, Högl B (2015). Quality control for diagnosis of REM sleep behavior disorder: criteria, questionnaires, video, and polysomnography. Disorders of sleep and circadian rhythms in Parkinson’s disease.

[17] Frauscher B, Mitterling T, Bode A (2014). A prospective questionnaire study in 100 healthy sleepers: non-bothersome forms of recognizable sleep disorders are still present. J Clin Sleep Med.

[18] Mahlknecht P, Seppi K, Frauscher B (2015). Probable RBD and association with neurodegenerative disease markers: a population-based study. Mov Disord.

[19] Stiasny-Kolster K, Sixel-Döring F, Trenkwalder C (2015). Diagnostic value of the REM sleep behavior disorder screening questionnaire in Parkinson’s disease. Sleep Med.

[20] Frauscher B, Högl B, Chokroverty S, Allen RP, Walters AS, Montagna P (2013). Sleep and movement disorders.

[21] Sforza E, Zucconi M, Petronelli R, Lugaresi E, Cirignotta F (1988). REM sleep behavioral disorders. Eur Neurol.

[22] Frauscher B, Gschliesser V, Brandauer E (2007). Video analysis of motor events in REM sleep behavior disorder. Mov Disord.

[23] Frauscher B, Gschliesser V, Brandauer E, Ulmer H, Poewe W, Högl B (2009). The relation between abnormal behaviors and REM sleep microstructure in patients with REM sleep behavior disorder. Sleep Med.

[24] Valli K, Frauscher B, Gschliesser V (2012). Can observers link dream content to behaviours in rapid eye movement sleep behaviour disorder? A cross-sectional experimental pilot study. J Sleep Res.

[25] Sixel-Döring F, Schweitzer M, Mollenhauer B, Trenkwalder C (2011). Intraindividual variability of REM sleep behavior disorder in Parkinson’s disease: a comparative assessment using a new REM sleep behavior disorder severity scale (RBDSS) for clinical routine. J Clin Sleep Med.

[26] Sixel-Döring F, Trautmann E, Mollenhauer B, Trenkwalder C (2014). Rapid eye movement sleep behavioral events: a new marker for neurodegeneration in early Parkinson disease?. Sleep.

[27] Ferri R, Marelli S, Cosentino FI, Rundo F, Ferini-Strambi L, Zucconi M (2013) Night-to-night variability of automatic quantitative parameters of the chin EMG amplitude (Atonia Index) in REM sleep behavior disorder. J Clin Sleep Med 9:253–258 10.5664/jcsm.2490 PMC3578682 23493642

[28] Zhang J, Lam SP, Ho CK, Li AM, Tsoh J, Mok V, Wing YK (2008) Diagnosis of REM sleep behavior disorder by video-polysomnographic study: is one night enough? Sleep 31:1179–1185 PMC2542964 18714790

[29] Frauscher B, Iranzo A, Högl B (2008). Quantification of electromyographic activity during REM sleep in multiple muscles in REM sleep behavior disorder. Sleep.

[30] Frauscher B, Iranzo A, Gaig C (2012). Normative EMG values during REM sleep for the diagnosis of REM sleep behavior disorder. Sleep.

[31] Lapierre O, Montplaisir J (1992). Polysomnographic features of REM sleep behavior disorder: development of a scoring method. Neurology.

[32] Iranzo A, Frauscher B, Santos H (2011). Usefulness of the SINBAR electromyographic montage to detect the motor and vocal manifestations occurring in REM sleep behavior disorder. Sleep Med.

[33] Schenck CH, Bundlie SR, Patterson AL, Mahowald MW (1987). Rapid eye movement sleep behavior disorder. A treatable parasomnia affecting older adults. JAMA.

[34] McCarter SJ, Louis StEK, Duwell EJ, Timm PC, Sandness DJ, Boeve BF, Silber MH (2014). Diagnostic thresholds for quantitative REM sleep phasic burst duration, phasic and tonic muscle activity, and REM atonia index in REM sleep behavior disorder with and without comorbid obstructive sleep apnea. Sleep.

[35] Frauscher B, Gabelia D, Biermayr M (2014). Validation of an integrated software for the detection of rapid eye movement sleep behavior disorder. Sleep.

[36] Ferri R, Manconi M, Plazzi G (2008). A quantitative statistical analysis of the submentalis muscle EMG amplitude during sleep in normal controls and patients with REM sleep behavior disorder. J Sleep Res.

[37] Ferri R, Rundo F, Manconi M (2010). Improved computation of the atonia index in normal controls and patients with REM sleep behavior disorder. Sleep Med.

[38] Schenck CH, Bundlie SR, Mahowald MW (1996). Delayed emergence of a parkinsonian disorder in 38% of 29 older men initially diagnosed with idiopathic rapid eye movement sleep behaviour disorder. Neurology.

[39] Iranzo A, Molinuevo JL, Santamaría J (2006). Rapid-eye-movement sleep behaviour disorder as an early marker for a neurodegenerative disorder: a descriptive study. Lancet Neurol.

[40] Postuma RB, Gagnon JF, Vendette M, Fantini ML, Massicotte-Marquez J, Montplaisir J (2009). Quantifying the risk of neurodegenerative disease in idiopathic REM sleep behavior disorder. Neurology.

[41] Schenck CH, Boeve BF, Mahowald MW (2013). Delayed emergence of a parkinsonian disorder or dementia in 81% of older men initially diagnosed with idiopathic rapid eye movement sleep behavior disorder: a 16-year update on a previously reported series. Sleep Med.

[42] Iranzo A, Tolosa E, Gelpi E (2013). Neurodegenerative disease status and post-mortem pathology in idiopathic rapid-eye-movement sleep behaviour disorder: an observational cohort study. Lancet Neurol.

[43] Mahlknecht P, Iranzo A, Högl B (2015). Olfactory dysfunction predicts early transition to a Lewy body disease in idiopathic RBD. Neurology.

[44] Postuma RB, Gagnon JF, Vendette M, Desjardins C, Montplaisir JY (2011) Olfaction and color vision identify impending neurodegeneration in rapid eye movement sleep behavior disorder. Ann Neurol 69:811–818 10.1002/ana.22282 21246603

[45] Scherfler C, Frauscher B, Schocke M (2011). White and gray matter abnormalities in idiopathic rapid eye movement sleep behavior disorder: a diffusion-tensor imaging and voxel-based morphometry study. Ann Neurol.

[46] Olson EJ, Boeve BF, Silber MH (2000). Rapid eye movement sleep behavior disorder: demographic, clinical and laboratory findings in 93 cases. Brain.

[47] Iranzo A, Lomeña F, Stockner H (2010). Decreased striatal dopamine transporter uptake and substantia nigra hyperechogenicity as risk markers of synucleinopathy in patients with idiopathic rapid-eye-movement sleep behaviour disorder: a prospective study. Lancet Neurol.

[48] Iranzo A, Valldeoriola F, Lomeña F (2011). Serial dopamine transporter imaging of nigrostriatal function in patients with idiopathic rapid-eye-movement sleep behaviour disorder: a prospective study. Lancet Neurol.

[49] Eisensehr I, Linke R, Noachtar S, Schwarz J, Gildehaus FJ, Tatsch K (2000). Reduced striatal dopamine transporters in idiopathic rapid eye movement sleep behavior disorder. Comparison with Parkinson’s disease and controls. Brain.

[50] Albin RL, Koeppe RA, Chervin RD (2000). Decreased striatal dopaminergic innervation in REM sleep behavior disorder. Neurology.

[51] Mahlknecht P, Pechlaner R, Boesveldt S et al (2016) Optimizing odor identification testing as quick and accurate diagnostic tool for Parkinson’s disease. Mov Disord. (in press) 10.1002/mds.26637 PMC5026160 27159493

[52] Iranzo A, Stockner H, Serradell M (2014). Five-year follow-up of substantia nigra echogenicity in idiopathic REM sleep behavior disorder. Mov Disord.

[53] Frauscher B, Jennum P, Ju YE (2014). Comorbidity and medication in REM sleep behavior disorder: a multicenter case-control study. Neurology.

[54] Postuma RB, Iranzo A, Hogl B (2015). Risk factors for neurodegeneration in idiopathic rapid eye movement sleep behavior disorder: a multicenter study. Ann Neurol.

[55] Howell MJ, Schenck CH (2015). Rapid eye movement sleep behavior disorder and Neurodegenerative disease. JAMA Neurol.

[56] McGrane IR, Leung JG, St Louis EK, Boeve BF (2015). Melatonin therapy for REM sleep behavior disorder: a critical review of evidence. Sleep Med.

[57] Cipolli C, Bolzani R, Massetani R, Murri L, Muratorio A (1992). Dream structure in Parkinson’s patients. J Nerv Ment Dis.

[58] Uguccioni G, Golmard JL, de Fontréaux AN, Leu-Semenescu S, Brion A, Arnulf I (2013). Fight or flight? Dream content during sleepwalking/sleep terrors vs. rapid eye movement sleep behavior disorder. Sleep Med.

[59] Valli K, Frauscher B, Gschliesser V (2012). Can observers link dream content to behaviours in rapid eye movement sleep behaviour disorder? A cross-sectional experimental pilot study. J Sleep Res.

[60] Valli K, Frauscher B, Peltomaa T, Gschliesser V, Revonsuo A, Högl B (2015). Dreaming furiously? A sleep laboratory study on the dream content of people with Parkinson’s disease and with or without rapid eye movement sleep behavior disorder. Sleep Med.

